# Virulence gene transcription, phylogroups, and antibiotic resistance of cervico-vaginal pathogenic *E*. *coli* in Mexico

**DOI:** 10.1371/journal.pone.0234730

**Published:** 2020-06-22

**Authors:** Eric Monroy-Pérez, Areli Bautista Cerón, Luis Rey García Cortés, Nancy Nolasco Alonso, Pablo Domínguez-Trejo, Tania Hernández-Jaimes, Jaime Bustos-Martínez, Aida Hamdan-Partida, Ernesto Arturo Rojas Jiménez, Sergio Vaca, Felipe Vaca-Paniagua, Gloria Luz Paniagua-Contreras

**Affiliations:** 1 Facultad de Estudios Superiores Iztacala, Universidad Nacional Autónoma de México, Tlalnepantla, Estado de México, México; 2 Instituto Mexicano del Seguro Social, Ciudad de México, México; 3 Departamento de Atención a la Salud, Universidad Autónoma Metropolitana Xochimilco, CDMX, México; 4 Laboratorio Nacional en Salud, Diagnóstico Molecular y Efecto Ambiental en Enfermedades Crónico-Degenerativas, Facultad de Estudios Superiores Iztacala, Universidad Nacional Autónoma de México, México City, México; 5 Unidad de Biomedicina, Facultad de Estudios Superiores Iztacala, Universidad Nacional Autónoma de México, México City, México; 6 Instituto Nacional de Cancerología, CDMX, México; University of Georgia, UNITED STATES

## Abstract

The pathogenicity of *Escherichia coli* strains that cause cervico-vaginal infections (CVI) is due to the presence of several virulence genes. The objective of this study was to define the variability regarding the genotype of antibiotic resistance, the transcription profiles of virulence genes after in vitro infection of the vaginal cell line A431 and the phylogroup composition of a group of cervico-vaginal *E*. *coli* strains (CVEC). A total of 200 *E*. *coli* strains isolated from Mexican women with CVI from two medical units of the Mexican Institute of Social Security were analysed. *E*. *coli* strains and antibiotic resistance genes were identified using conventional polymerase chain reaction (PCR), and phylogroups were identified using multiplex PCR. Virulence gene transcription was measured through reverse-transcriptase real-time PCR after infection of the vaginal cell line A431. The most common antibiotic resistance genes among the CVEC strains were *aac(3)II*, TEM, *dfrA1*, *sul1*, and *qnrA*. The predominant phylogroup was B2. The genes most frequently transcribed in these strains were *fimH*, *papC*, *irp2*, *iroN*, *kpsMTII*, *cnf1*, and *ompT*, mainly in CVEC strains isolated from chronic and occasional vaginal infections. The strains showed a large diversity of transcription of the virulence genes phenotype and antibiotic resistance genotype, especially in the strains of phylogroups, B2, A, and D. The strains formed 2 large clusters, which contained several subclusters. The genetic diversity of CVEC strains was high. These strains have a large number of transcription patterns of virulence genes, and one-third of them carry three to seven antibiotic resistance genes.

## Introduction

Vaginal colonisation by *Escherichia coli* causes several genitourinary diseases, including pelvic inflammatory disease, urinary tract infection [1], and neonatal meningitis during pregnancy [2]. The frequency of aerobic cervicovaginal infections (CVI) in women of reproductive age caused by cervico-vaginal *E*. *coli* (CVEC) is 11% [3]. Transmission of *E*. *coli* in CVI has been shown to be due to the anatomical proximity of the "anorectal/vagina" region [4]. The pathogenicity of *E*. *coli* is due to several virulence factors including adhesins, iron acquisition systems, toxins, and protectins [5]. The genes encoding these virulence factors are often found in pathogenicity islands, which can be transferred horizontally by transposons, bacteriophages, or plasmids [6]. Virulence genes of *E*. *coli* promote bacterial colonisation, host tissue damage [7], biofilm formation, and immune evasion [8]. Pathogenic *E*. *coli* strains are classified into eight phylogroups, seven belonging to *E*. *coli* sensu stricto (A, B1, B2, C, D, E, and F), and one belonging to the *Escherichia* cryptic clade I [9]. The development of urinary tract-infecting *E*. *coli* strains resistant to several families of antibiotics is a serious health problem that reduces treatment efficacy [10,11]. There have been few studies on the transcription of CVEC virulence genes *in vivo* and *in vitro*. The objective of this study was to simulate the conditions of a vaginal infection caused by clinical isolates *E*. *coli* to determine the variability regarding the genotype of antibiotic resistance, the transcription profiles of virulence genes after *in vitro* infection of the vaginal cell line A431 and the phylogroup composition of a group of CVI-causing *E*. *coli* strains.

## Materials and methods

### Isolation of *E*. *coli* strains from patients

This study included 210 women attending to medical consultation in the Family Medical Units 64 and 62 of the Mexican Social Security Institute (Instituto Mexicano del Seguro Social–IMSS, Tlalnepantla, Estado de Mexico) (aged 18 to 69 years) with signs and symptoms of CVI (vaginal discharge, inflammation, and pain), from September 2016 to January 2017. All women acquired the CVIs outside the Family Medical Units. The infections were grouped into three categories; occasional vaginal infection (one sporadic episode), recurrent (4 or more episodes per year) or chronic (persistent infection over time despite antibiotic treatment). The study participants reported not having been treated with antibiotics in the past three months and signed an informed consent form. Research ethics committee of the Family Medical Units 64 and 62 of the IMSS approved the study. The physicians collected the samples from the cervical wall using sterile swabs and a vaginal mirror. The whole sterile swabs were transferred to brain heart infusion (BHI) broth (Dibico Laboratories, Mexico) and incubated at 37 °C for 24 h. After that, the cultures were resuspended using a vortex, and the swab with the culture samples were seeded on solid agar plates of eosin-methylene blue (EMB) medium (Bioxon, Mexico) and incubated at 37 °C for 24 h. The *E*. *coli* strains were identified by standard IMViC biochemical tests (Indole, Methyl Red, Voges-Proskauer and Citrate) and polymerase chain reaction (PCR) by amplification and sequencing of the 16S rRNA gene as described elsewhere [12]. *E*. *coli* ATCC 11775 was used as a positive control in each PCR assay. In all the bacterial cultures of the patients were confirmed that *E*. *coli* was the cause of CVI.

### Identification of antibiotic resistance genes

The primers and PCR conditions used to amplify the beta-lactamase genes TEM and CITM were described by Dallenne et al. [13] and Van et al. [14], respectively (Table 1); whereas the primers and conditions to amplify the genes that confer resistance to chloramphenicol (*cmlA*), tetracycline (*tetA*), quinolones (*qnrA*), sulfamethoxazole (*sul1*), and trimethoprim (*dfrA1*) were described by Momtaz et al. [15], and for the genes that confer resistance to aminoglycosides (*aac(3)II*) by Sáenz et al. [16]. For each singleplex PCR assay the final volume per reaction mixture was 25 μL; 12.5 μL of Taq DNA Polymerase 2x Master Mix RED (Ampliqon), 1μL of each forward and reverse primers (10 pmol, Integrated DNA Technologies), 7.5 μL of nuclease-free water and 3 μL of DNA template (100 ng).

**Table 1:** Primers used for detection of antimicrobial resistant genes in *Escherichia coli*.

Antibiotic	Gen	Sequence (5´-3´)	PCR product size (bp)
Beta-lactams	TEM	(F) CATTTCCGTGTCGCCCTTATTC (R) CGTTCATCCATAGTTGCCTGAC	800
	CITM	(F) TGGCCAGAACTGACAGGCAAA (R) TTTCTCCTGAACGTGGCTGGC	462
Chloramphenicol	*cmlA*	(F) CCGCCACGGTGTTGTTGTTATC (R) CACCTTGCCTGCCCATCATTAG	698
Tetracycline	*tet(A)*	(F) GGTTCACTCGAACGACGTCA (R) CTGTCCGACAAGTTGCATGA	577
Quinolones	*qnr*	(F) GGGTATGGATATTATTGATAAAG (R) CTAATCCGGCAGCACTATTTA	670
Sulfonamide	*sul1*	(F) TTCGGCATTCTGAATCTCAC (R) ATGATCTAACCCTCGGTCTC	822
Trimethoprim	*dfrA1*	(F) GGAGTGCCAAAGGTGAACAGC (R) GAGGCGAAGTCTTGGGTAAAAAC	367
Aminoglycosides	*aac(3)II*	(F) ACTGTGATGGGATACGCGTC (R) CTCCGTCAGCGTTTCAGCTA	237

### Detection of phylogroups

A multiplex PCR method was used to identify the phylogroups A, B1, B2, C, D, E, F, and cryptic clade I, as previously described by Clermont et al. [9]. This approach allowed to amplify *chuA*, *yjaA*, the DNA fragment designated as *TspE4*.*C2* and *arpA* to identify the different phylogroups (Table 2). The final multiplex PCR reaction volume was 20 μL; 9 μL of Taq DNA Polymerase 2x Master Mix RED (Ampliqon), 1 μL of first forward and reverse primers and 3 μL of DNA template (100 ng). To determine groups E and C a singleplex PCR was used separately. For groups E and C an internal control was used (Table 2). The PCR conditions were as follows: 4 min at 94 °C, 30 cycles of 5 s at 94 °C and 20 s at 57 °C (group E) or 59 °C (group C) and a final extension of 5 min at 72 °C.

**Table 2:** Primers used for detection of phylogroups in *Escherichia coli*.

PCR reaction	Target	Sequence (5´-3´)	PCR product size (bp)
Multiplex	*chuA*	(F) ATGGTACCGGACGAACCAAC (R) TGCCGCCAGTACCAAAGACA	288
	*yjaA*	(F) CAAACGTGAAGTGTCAGGAG (R) AATGCGTTCCTCAACCTGTG	211
	*TspE4*.*C2*	(F) CACTATTCGTAAGGTCATCC. (R) AGTTTATCGCTGCGGGTCGC	152
	*arpA*	(F) AACGCTATTCGCCAGCTTGC (R) TCTCCCCATACCGTACGCTA	400
Group E	*arpA*	(F) GATTCCATCTTGTCAAAATATGCC (R) GAAAAGAAAAAGAATTCCCAAGAG	301
Group C	*trpA*	(F) AGTTTTATGCCCAGTGCGAG (R) TCTGCGCCGGTCACGCCC	219
Internal control	*trpA*	(F) CGGCGATAAAGACATCTTCAC (R) GCAACGCGGCCTGGCGGAAG	489

### Cell line infection

The human vaginal cell line A431 (ATCC, Manassas, VA, USA) was used as an *in vitro* model of infection with CVEC strains to determine the transcription of virulence genes. This model is frequently used to study host-pathogen interactions [17,18]. Each *E*. *coli* strain was seeded in BHI broth and incubated at 37 °C for 12 h under constant stirring. Following the instructions of the RNAprotect Bacteria Reagent Handbook (Qiagen), each *E*. *coli* strain was seeded in BHI broth and incubated at 37 °C for 12 h under constant stirring. The bacterial culture was diluted 1:4 using phosphate-buffered saline and the bacterial concentration was estimated by optical density at 600 nm in a Beckman DU-7400 spectrophotometer (optical density of 600 nm = 1.0 corresponded to 1x10^9^ cells/mL). From this preparations, dilutions were made to obtain a concentration of 2 x 10^6^ cells/mL. In 24-well plates, fifty microliters of the dilution were inoculated on the surface of a 180,000 A431 cell monolayer and incubated with 1 mL of F12K plus 10% bovine foetal serum at 37 °C for 72 h in a 5% CO_2_ atmosphere and saturated humidity. After incubation A431 cells reach full confluency with ~250,000 cells. This represents a multiplicity of infection of 8 *E*. *coli* per A431 cell. The maintenance medium (F12K plus 10% bovine foetal serum) was changed every 24 h. Each experiment was done in triplicate.

### Purification of *E*. *coli* RNA and reverse transcription

RNA purification was performed in a QIAcube robotic workstation (Qiagen, Hilden, Germany) using the commercial kit RNeasy Mini Kit (Qiagen), which involved bacterial lysis with TE buffer (10 mM Tris-HCl, 1 mM EDTA, pH 8) containing 1 mg/mL of lysozyme. Each *E*. *coli* strain was harvested from the surface of the A431 cell culture, suspended in 1,000 μL of RNA Protect Bacteria Reagent (Qiagen), and vortexed for 30 s. The samples were centrifuged at 8000 x g for 10 min to obtain a bacterial cell pellet. The concentration and purity of total RNA were determined using a NanoDrop 2000 spectrophotometer (Thermo Fisher Scientific, Santa Clara CA, USA). The first cDNA strand synthesis was carried out using the Reverse Transcription QuantiTect kit (Qiagen) following the manufacturer’s instructions.

### Amplification of the virulence genes of *E*. *coli* by real-time PCR

The primers used to determine the transcription of the *E*. *coli* virulence genes encoding adhesins (*fimH* [type-1 fimbriae], *papA* and *papC* [pilus associated with pyelonephritis], *sfa* [S fimbriae], *afa* [afimbrial adhesin] and *focG* [fimbria F1C]) (Table 3), and genes encoding iron acquisition systems [*iroN* (iron) and *irp2* (iron-repressible protein)], protectins (*iss* [increased serum-survival protein] and *kpsMTII* [K-antigen]), toxins (*cnf1* [cytotoxic necrotising factor 1] and *astA* [enteroaggregative heat-stable toxin]), and other proteins (*ompT* [outer-membrane protease T]) were described by Johnson et al. [19] and Momtaz et al. [15].

**Table 3:** Primers used to determine the transcription of virulence genes in *Escherichia coli*.

Function	Gen	Sequence (5´-3´)
Adhesins	*fimH*	(F) TGCAGAACGGATAAGCCGTGG (R) GCAGTCACCTGCCCTCCGGTA
	*papA*	(F) ATGGCAGTGGTGTTTTGGTG (R) CGTCCCACCATACGTGCTCTTC
	*papC*	(F) GTGGCAGTATGAGTAATGACCGTTA (R) ATATCCTTTCTGCAGGGATGCAATA
	*sfa*	(F) GTGGATACGACGATTACTGTG (R) CCGCCAGCATTCCCTGTATTC
	*afa*	(F) GCTGGGCAGCAAACTGATAACTCTC (R)CATCAAGCTGTTTGTTCGTCCGCCG
	*focG*	(F) CAGCACAGGCAGTGGATACGA (R) GAATGTCGCCTGCCCATTGCT
Iron acquisition	*iroN*	(F) AAGTCAAAGCAGGGGTTGCCCG (R) GACGCCGACATTAAGACGCAG
	*irp2*	(F) AAGGATTCGCTGTTACCGGAC (R) AACTCCTGATACAGGTGGC
Protectins	*iss*	(F) ATCACATAGGATTCTGCCG (R) CAGCGGAGTATAGATGCCA
	*kpsMTII*	(F) GCGCATTTGCTGATACTGTTG (R) CATCCAGACGATAAGCATGAGCA
Toxins	*cnf1*	(F) AAGATGGAGTTTCCTATGCAGGAG (R) TGGAGTTTCCTATGCAGGAG
	*astA*	(F) ATGCCATCAACACAGTATAT (R) GCGAGTGACGGCTTTGTAGT
Other proteins	*ompT*	(F) ATCTAGCCGAAGAAGGAGGC (R) CCCGGGTCATAGTGTTCATC

Each Real-time PCR assay was done in 25 μL, and included 12.5 μL of the Master mix of the Rotor-Gene SYBR Green PCR kit (Qiagen), 1 μL of each forward and reverse primers (1 μM), 2μL of the cDNA (100 ng) and 8.5μL H_2_O of RNase-free water. Amplification was carried out at 95 °C for 5 min, followed by 40 cycles of 95 °C for 5 s and extension at 60 °C for 10 s. The threshold for the Ct definition was calculated with the Rotor-Gene Q 5plex HRM System software version 2.3.1.49 (Qiagen). Each real-time PCR assay included a melting curve, the housekeeping genes (*rpoE* y *arcA*), and 7 *E*. *coli* uropathogenic strains harbouring (EC112, EC101, EC131, EC144, EC153, EC160 and EC199) harbouring 13 virulence genes (*fimH*, *papC*, *papA*, *sfa*, *focG*, *afa*, *kpsmTII*, *iss*, *irp2*, *iroN*, *cnf1*, *astA* and *ompT*), previously described by us, were used as positive controls [20,21]. No template reactions were used as negative controls.

To establish the differences between the transcription percentages of the *E*. *coli* virulence genes associated with the vaginal infection diagnoses (occasional, recurrent and chronic) the Chi-square test was used with the SPSS statistical program with p <0.05 as significant.

### Unsupervised hierarchical clustering

To systematically categorize the strains by the genotype for antibiotic resistance genes and their transcription pattern of virulence genes (phenotype), we used unsupervised hierarchical clustering with the Gower similarity algorithm for categoric variables [22]. A matrix of categorical data, including phylogroup and antibiotic resistance genes and virulence genes transcription, as well as infection type (occasional, recurrent, and chronic) was performed in R (v3.6.1) with the cluster (2.1.0) package. The distance of each strain was calculated based on the general coefficient of similarities, which estimates the maximum possible absolute discrepancy between each combination pair of strains. With the calculated distances, mutually exclusive groups were clustered with the Ward’s method using R [23]. The strains were visualized in a genotype-phenotype distribution plot with a dendrogram done with the program hclust (v3.6.2, R core).

## Results

*E*. *coli* was identified through PCR in 95.2% (n = 200) of the evaluated patients. Occasional vaginal infection was more frequent among women studied (n = 149), with respect to recurrent (n = 20) and chronic (n = 31) infection (Table 4). The most common antibiotic resistance genes in CVEC strains were the aminoglycoside *aac(3)II* (82.5%), the beta-lactam TEM (61.5%), *dfrA1* (trimethoprim, 40.5%), *sul1* (sulfamethoxazole, 35.5%), and *qnrA* (quinolone 34.5%). Most strains isolated from chronic vaginal infections were carriers of the TEM, *tetA*, and *aac(3)II* genes, while in the recurrent infections were *aac(3)II* and *dfrA1* and in occasional infections *aac(3)II* and TEM (Table 4). In addition, 66% (n = 132) of the strains harboured 3 to 7 antibiotic resistance genes (Table 4). The number of multiple carrier strains of 4, 5 and 6 antibiotic resistance genes was higher in chronic infections than in recurrent infection.

**Table 4:** Frequencies of antibiotic resistance genes in CVEC strains.

Antibiotics	Gene	Infection (No. of strains)			Total number of strains n = 200 (%)
		Occasional vaginal n = 149 (%)	Recurrent vaginal n = 20 (%)	Chronic vaginal n = 31 (%)	
Betalactams	TEM	93 (62.4)	9 (45)	21 (67.7)	123 (61.5)
	CITM	19 (12.7)	4 (20)	6 (19.3)	29 (14.5)
Chloramphenicol	*cmlA*	7 (4.7)	0 (0)	2 (6.4)	9 (4.5)
Tetracycline	*tetA*	82 (55)	10 (50)	20 (64.5)	112 (56)
Quinolone	*qnrA*	53 (35.6)	7 (35)	9 (29.8)	69 (34.5)
Sulfamethoxazole	*sul1*	54 (36.2)	3 (15)	14 (45.1)	71 (35.5)
Trimethoprim	*dfrA1*	55 (36.9)	12 (60)	14 (45.1)	81 (40.5)
Aminoglycoside	*aac(3)II*	122 (81.9)	17 (85)	26 (83.9)	165 (82.5)
Number of resistance genes in strains	0	1 (0.7)	0 (0)	0 (0)	1 (0.5)
	1	19 (12.7)	3 (15)	5 (16.1)	27 (13.5)
	2	33 (22.1)	5 (25)	2 (6.4)	40 (20)
	3	38 (25.5)	3 (15)	3 (9.7)	44 (22)
	4	29 (19.5)	5 (25)	10 (32.2)	44 (22)
	5	21 (14.1)	3 (15)	5 (16.1)	29 (14.5)
	6	9 (6)	1 (5)	2 (6.4)	12 (6)
	7	2 (1.3)	0 (0)	1 (3.2)	3 (1.5)

The most frequent phylogroups in the CVEC strains were B2, A, D, and C, whereas the least common phylogroups were B1, F, and cryptic clade I (Table 5). The predominant phylogroup in all the CVEC strains was B2. The predominant phylogroups in CVEC from chronic infections were A and C, while those from the occasional infections were B2 and D (Table 5).

**Table 5:** Distribution of the different phylogroups in CVEC strains.

Phylogenetic groups	Infection (No. of strains)			Total number of strains n = 200 (%)
	Occasional vaginal n = 149 (%)	Recurrent vaginal n = 20 (%)	Chronic vaginal n = 31 (%)	
A	18 (12)	3 (15)	5 (16.1)	26 (13)
B1	8 (5.3)	0 (0)	2 (6.4)	10 (5)
B2	69 (46.3)	10 (50)	14 (45.1)	93 (46.5)
C	10 (6.7)	2 (10)	4 (12.9)	16 (8)
D	20 (13.4)	1 (5)	1 (3.2)	22 (11)
E	0 (0)	0 (0)	0 (0)	0 (0)
F	4 (2.7)	0 (0)	0 (0)	4 (2)
CLADE I	4 (2.7)	3 (15)	1 (3.2)	8 (4)
Not detected	16 (10.7)	1 (5)	4 (12.9)	21 (10.5)

After *in vitro* infection of the A431 cell line, the most frequently transcribed genes were *fimH* (89.5%) *kpsmTII* (72.5%), *irp2* (58.5%), and *cnf1* (33.5%). These genes were transcribed mainly in CVEC strains isolated from occasional vaginal infections and chronic vaginal. The less frequently transcribed genes were *astA*, *afa*, and *iss* (Table 6). CVEC strains isolated from chronic infections transcribed more virulence genes in relation to recurrent infection strains. Transcription of most genes (*fimH*, *papC*, *papA*, *sfa*, *focG*, *afa*, *kpsmTII*, *iss*, *iroN*, *cnf1*, *astA*, and *ompT*) was not associated with any of the three diagnoses (occasional, recurrent and cronic); however *irp2* was significantly associated with occasional vaginal infection (Chi-square test p <0.05, Table 6).

**Table 6:** Transcription of virulence genes in CVEC strains (n = 200).

Function	Gene	Infection (No. of strains)			Total number of strains (n = 200) %	p value*
		Occasional vaginal (n = 149)	Recurrent vaginal (n = 20)	Chronic vaginal (n = 31)		
Adhesins	*fimH*	128 (85.9)	17 (85)	28 (90.3)	173 (89.5)	NS
	*papC*	46 (30.8)	2 (10)	10 (32.2)	58 (29)	NS
	*papA*	22 (14.7)	2 (10)	4 (12.9)	28 (14)	NS
	*sfa*	17 (11.4)	0 (0)	3 (9.6)	20 (10)	NS
	*focG*	9 (6)	0 (0)	3 (9.6)	12 (6)	NS
	*afa*	5 (3.3)	1 (5)	1 (3.2)	7 (3.5)	NS
Protectins	*kpsmTII*	107 (71.8)	15 (75)	23 (74.2)	145 (72.5)	NS
	*iss*	6 (4)	1 (5)	1 (3.2)	8 (4)	NS
Iron acquisition	*irp2*	**97 (65)**	7 (35)	13 (41.9)	117 (58.5)	**0.005**
	*iroN*	49 (32,8)	4 (20)	12 (38.7)	65 (32.5)	NS
Toxins	*cnf1*	48 (32.2)	7 (35)	12 (38.7)	67 (33.5)	NS
	*astA*	4 (2.7)	0 (0)	0 (0)	4 (2)	NS
Other proteins	*ompT*	36 (24.1)	3 (15)	9 (29)	48 (24)	NS

*Chi-square test, p values (for a three-group comparison) are shown only when significant (p <0.05). NS: not significant.

**Fig 1:**
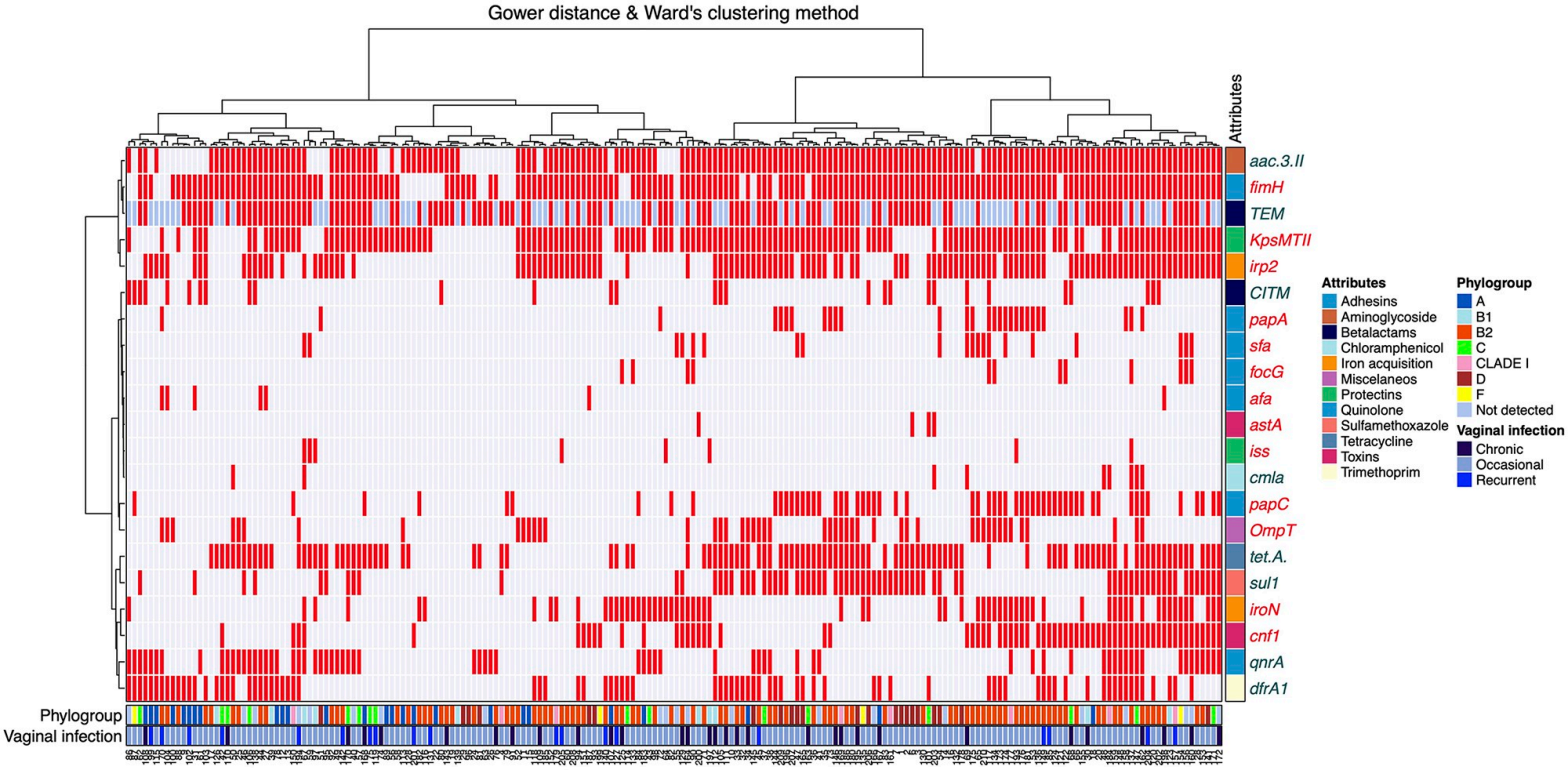
Patterns of transcription of virulence genes and antibiotic resistance genes genotypes in the phylogroups of CVEC strains. The 200 strains were classified and clustered according to their virulence genes transcription (phenotype) and for the presence of antibiotic resistance genes (genotype). The positivity for a given phenotype or genotype is depicted with a red rectangle, gray is used for negativity and blue for inconclusive determination for the TEM gene. The phylogroups and the gene function are color coded as shown in the legend. Cladograms for the strains and genes are shown on top and the left side. Type of infection (occasional, recurrent and chronic) is shown in the lower panel.

To better characterize the distribution of the CVEC strains according to their composition of antibiotic resistance genotype and their virulence gene transcription phenotype, we performed a clustering analysis (see materials and methods). The strains showed a marked heterogeneity, characterized by a broad distribution of the positivity for the genotypes and phenotypes. There were two large clusters divided mainly by the positivity for TEM, *fimH*, *aac*.*3*.*II*, *KpsMTIII*, *irp2*, *tetA* and *sul1* (Fig 1). Cluster 1 was composed by 107 strains and group 2 by 93. Cluster 1 was more diverse in phylogroup composition, being B2 (43/107) and A (22/107) the most represented. Cluster 2 was more homogeneous and characterized by a large fraction of B2 strains (50/93), followed by phylogroup D strains (15/93). In addition, genes that mostly grouped together included TEM, *fimH*, *aac*.*3*.*II* and *KpsMTIII; irp2*, *tetA* and *sul1; dfrA1* and *qnRA; and cnf1* and *iroN* (Fig 1). We found no obvious grouping of the strains by their genotype, virulence phenotype, phylogroup or the type of infection, which highlights the notable biological heterogeneity of these strains.

## Discussion

In this study, we performed molecular analysis of a group of *E*. *coli* strains (n = 200) collected from women with signs and symptoms of CVI. The majority of *E*. *coli* strains were isolated from women with occasional infection, in relation to recurrent and chronic infection (Table 4). Persistent vaginal colonisation by *E*. *coli* is a significant risk factor for acute cystitis, recurrent urinary tract infections [24], and premature rupture of membranes, which lead to preterm labour [25]. The frequency of *E*. *coli* strains (95.2%) in the study sample was higher than that found in other countries [26,27].

The increase in the frequency of genitourinary strains of *E*. *coli* resistant to several groups of antibiotics is a significant health problem that limits the treatment of infections [28]. In this study, most CVEC strains (n = 132) were carriers of 3 to 7 antibiotic resistance genes, mainly in strains associated with chronic infections, and the most common ones were *aac(3)II* (aminoglycosides), TEM (beta-lactams), *tetA* (tetracycline), *dfrA1* (trimethoprim), *sul1* (sulfamethoxazole), and *qnrA* (quinolones) (Table 4). This result agrees with the high rates of resistance to ampicillin (94.6%), tetracycline (92.4%), nalidixic acid (88.6%), gentamicin (77.2%), amikacin (68.9%), and trimethoprim-sulfamethoxazole (67.4%) previously identified in CVEC strains [5]. The frequency of the genotype of resistance to beta-lactams, trimethoprim, sulfamethoxazole, aminoglycosides and quinolone coincides with the resistance phenotype, evaluated by the Kirby-Bauer method, recently published by our working group for these same CVEC strains [29]. The frequencies of the genes *aac(3)II*, *tetA*, and *dfrA1* in the evaluated *E*. *coli* strains were higher than those in uropathogenic strains of *E*. *coli* in Iran, whereas the frequencies of the genes *qnrA* and *sul1* were similar to those in these strains [15]. In contrast, the frequency of the gene TEM, was similar to that in *E*. *coli* strains identified in other parts of the world [30,31]. The bacterial horizontal transfer of antimicrobial resistance genes by means of plasmids, transposons, and integrons favours the dissemination of multiresistant strains [32]. In Mexico, the rate of multidrug resistance to antibiotics in strains causing CVI has not been studied much. However, one study found that the rate of resistance to cephalothin, ampicillin, carbenicillin, pefloxacin, cefotaxime, and trimethoprim-sulfamethoxazole was high in uropathogenic strains of *E*. *coli* [20]. Overall, the detection rates of the TEM, *cmlA*, *tetA* and *sul1* genes were higher in CVEC strains from chronic infections, while CITM, *qnrA*, *dfrA1* and *aac(3)II* were more frequent in strains from recurrent infections (Table 4). In Mexico, a previous study reported the prevalence of the clone O25-ST131, associated with community-acquired urinary tract infections that carry determinants for extended-spectrum β-lactamase (ESBLs), trimethoprim-sulfamethoxazole and fluoroquinolones resistance [33]. It is probable that these determinants could be transferred to vaginal strains, which would explain the high frequency of TEM and *sul1* in strains isolated from patients with occasional vaginal infection (Table 4). In addition, the fact that *dfrA1* was more frequent in strains of patients with recurrent vaginal infection could be attributed that in Mexico trimethoprim has been used relatively frequently to treat different episodes of recurrent urinary infections, while to treat chronic infections. other antibiotics have been used [34].

There are not many studies on the transcription of virulence genes by *E*. *coli* strains during vaginal infections. For this reason, an *in vitro* vaginal infection model with cell line A431 was used to determine the qualitative transcription of genes encoding adhesins, iron acquisition systems, protectins, and toxins in CVEC strains for 72 h. The infection period of 72 h was established to determine the transcription of genes at the beginning of infection and those that are likely to be transcribed during the more advanced infection. In this study, the most commonly transcribed genes were *fimH* (89.5%) and *papC* (29%) (cellular adhesion); *irp2* (58.5%) and *iroN* (32.5%) (iron acquisition systems), *kpsMTII* (72.5%) (protectins); and *cnf1* (33.5%) (toxins) (Table 6), which were mainly observed in CVEC strains isolated from chronic and occasional infections. These results are relevant because they demonstrate, for the first time, the combined transcription of different virulence genes during infection of vaginal cells. The frequencies of transcription of the genes *fimH*, *irp2*, *iroN*, *kpsMTII*, and *cnf1* were higher than the rates previously found by our research group in uropathogenic *E*. *coli* strains *in vitro*, except for the *papC* gene, which had a similar frequency [35]. It has been demonstrated that the expression of *fim* in *E*. *coli* promotes bacterial colonisation, invasion, and biofilm formation [36], whereas the presence of *pap* has been associated with pyelonephritis and recurrent urinary tract infections in women [37]. The expression of the iron acquisition genes *irp2* and *iroN* in strains causing CVI is associated with bacterial survival and multiplication during infection of the vaginal cell line because iron is an essential element that facilitates electron transport and nucleotide synthesis and reduces peroxide [38]. The high transcription of the capsular antigen *kpsMTII* (n = 145, Table 6) demonstrates the capacity of CVEC strains to cause chronic infections because the capsule is essential for protection against complement-mediated killing and phagocytosis [39]. The transcription percentages of most of the CVEC genes were not found to be associated with any of the three clinical diagnoses (Table 6), except for *irp2*, which was associated with occasional infection, suggesting that pathogenesis during occasional, recurrent and chronic infection caused by CVEC strains is very similar.

In this study, we identified a large diversity of transcription of the virulence genes phenotype and antibiotic resistance gene genotype, especially in the strains of phylogroups, B2, A, and D associated to the three types of infection (Fig 1). The strains formed 2 large clusters, which contained several subclusters. Most subclusters were observed in phylogroup B2 and were formed by genes *fimH*, *irp2*, and *kpsMTII* in association with the antibiotic resistance genes TEM, *tetA*, *dfrA1*, and *aac(3)II*. The characteristics of the phylogroup B2 in *E*. *coli* strains are similar to those of vaginal *E*. *coli* strains reported in others parts of the world, including high frequencies of the genes *fimH*, *irp2*, *ompT*, *iroN*, and antibiotic resistance genes [5]. We could not identify the phylogroup of 21 strains, possibly due to genetic recombination events or variation at the recognition sites of the primers, which may have prevented amplification (Table 5) [40]. It is also possible that these strains belong to very rare phylogroups that are not covered by this method [7]. In addition, the clustering analysis showed a notable biological heterogeneity of the strains at expression, genotype and phylogroup levels. Furthermore, chronic and occasional infections were almost evenly distributed among the two large groups. While our strategy to group the strains was sound and systematic, the large diversity of the strains prevented an obvious clustering of biological or clinical characteristics. There are relatively few reports in the literature that characterize CVEC strains, and they report a variable level of heterogeneity, and a lack of association with clinical variables [5,25,26].

The detection of different transcription patterns of virulence genes during *in vitro* infection demonstrates the virulence of the strains due to their capacity for cell adhesion, multiplication, internalisation, and evasion of the immune response. We hypothesize that this may lead to chronic and/or acute infections, and may also promote recurrent infections of the urinary tract, especially because of the presence of multiple antibiotic resistance genes. This is the first study conducted in Mexico on the expression patterns of virulence genes and their association with antibiotic resistance genes and phylogroups in CVEC strains, and the results may serve to guide the correct prescription of antibiotics for treating this class of infections.
